# Association of Maternal Preeclampsia With Offspring Risks of Ischemic Heart Disease and Stroke in Nordic Countries

**DOI:** 10.1001/jamanetworkopen.2022.42064

**Published:** 2022-11-15

**Authors:** Fen Yang, Imre Janszky, Mika Gissler, Nathalie Roos, Anna-Karin Wikström, Yongfu Yu, Hua Chen, Anna-Karin Edstedt Bonamy, Jiong Li, Krisztina D. László

**Affiliations:** 1 Department of Global Public Health, Karolinska Institutet, Stockholm, Sweden; 2 Department of Public Health and Nursing, Norwegian University of Science and Technology, Trondheim, Norway; 3 Department of Knowledge Brokers, Finnish Institute for Health and Welfare, Helsinki, Finland; 4 Academic Primary Health Care Centre, Region Stockholm, Stockholm, Sweden; 5 Department of Molecular Medicine and Surgery, Karolinska Institutet, Stockholm, Sweden; 6 Division of Clinical Epidemiology, Department of Medicine Solna, Karolinska University Hospital, Karolinska Institutet, Stockholm, Sweden; 7 Department of Women’s and Children’s Health, Uppsala University, Uppsala, Sweden; 8 Department of Biostatistics, School of Public Health, and The Key Laboratory of Public Health Safety of Ministry of Education, Fudan University, Shanghai, China; 9 Department of Clinical Epidemiology, Aarhus University Hospital, Aarhus, Denmark

## Abstract

**Importance:**

An association between maternal preeclampsia and an increased risk of cardiovascular disease in the offspring is plausible, but evidence in this area is limited.

**Objective:**

To investigate (1) the association between maternal preeclampsia and risks of ischemic heart disease (IHD) and stroke in the offspring, (2) whether the association varies by severity or timing of onset of preeclampsia, and (3) the role of preterm birth and small for gestational age (SGA) birth, both of which are related to preeclampsia and cardiovascular diseases, in this association.

**Design, Setting, and Participants:**

This multinational population-based cohort study obtained data from Danish, Finnish, and Swedish national registries. Live singleton births from Denmark (1973-2016), Finland (1987-2014), and Sweden (1973-2014) were followed up until December 31, 2016, in Denmark and December 31, 2014, in Finland and Sweden. Data analyses were performed between September 2020 and September 2022.

**Exposures:**

Preeclampsia and its subtypes, including early onset (<34 gestational weeks) and late onset (≥34 gestational weeks), severe and mild or moderate, and with and without SGA birth.

**Main Outcomes and Measures:**

Diagnoses of IHD and stroke were extracted from patient and cause-of-death registers. Cox proportional hazards regression models and flexible parametric survival models were used to analyze the associations. Sibling analyses were conducted to control for unmeasured familial factors.

**Results:**

The cohort included of 8 475 819 births (2 668 697 [31.5%] from Denmark, 1 636 116 [19.3%] from Finland, and 4 171 006 [49.2%] from Sweden, comprising 4 350 546 boys [51.3%]). Of these offspring, 188 670 (2.2%) were exposed to maternal preeclampsia, 7446 (0.1%) were diagnosed with IHD, and 10 918 (0.1%) were diagnosed with stroke during the median (IQR) follow-up of 19.3 (9.0-28.1) years. Offspring of individuals with preeclampsia had increased risks of IHD (adjusted hazard ratio [HR], 1.33; 95% CI, 1.12-1.58) and stroke (adjusted HR, 1.34; 95% CI, 1.17-1.52). These associations were largely independent of preterm or SGA birth. Severe forms of preeclampsia were associated with a higher stroke risk than less severe forms (severe vs mild or moderate: adjusted HR, 1.81 [95% CI, 1.41-2.32] vs 1.22 [95% CI, 1.05-1.42]; early vs late onset: adjusted HR, 2.55 [95% CI, 1.97-3.28] vs 1.18 [95% CI, 1.01-1.39]; with vs without SGA birth: adjusted HR, 1.84 [95% CI, 1.44-2.34] vs 1.25 [95% CI, 1.07-1.48]). Sibling analyses suggested that the associations were partially explained by unmeasured familial factors.

**Conclusions and Relevance:**

Results of this study suggest that offspring born to individuals with preeclampsia had increased IHD and stroke risk that were not fully explained by preterm or SGA birth, and that the associated risks for stroke were higher for severe forms of preeclampsia.

## Key Points

**Question:**

Is maternal preeclampsia associated with increased risks of ischemic heart disease (IHD) and stroke in the offspring?

**Findings:**

In this cohort study involving almost 8.5 million participants in Nordic countries, offspring who were prenatally exposed to maternal preeclampsia had a 33% increased risk of IHD and a 34% increased risk of stroke in childhood and young adulthood; these associations were not fully explained by preterm or small for gestational age birth. The associated risk of stroke was higher for severe than for milder forms of preeclampsia and persisted in the sibling analyses.

**Meaning:**

Findings of this study suggest that maternal preeclampsia was associated with increased risks of IHD and stroke in the offspring.

## Teaser

This cohort study examines the risk of cardiovascular diseases among offspring who had prenatal exposure to severe or mild forms of preeclampsia.

## Introduction

Cardiovascular diseases (CVDs) are responsible for approximately one-third of deaths worldwide and represent a huge economic burden.^1^ Among CVDs, ischemic heart disease (IHD) and stroke are the 2 main causes of death.^2^ There is a stable or an increasing incidence of IHD and stroke in populations younger than 50 years.^3,4^ Traditional cardiovascular risk factors, such as smoking, dyslipidemia, and hypertension,^4,5^ do not fully explain IHD and stroke in young people^6,7^; thus, further investigation of novel risk factors is warranted.

Preeclampsia, a major placenta-related complication, affects approximately 3% to 5% of pregnancies worldwide.^8^ Although the associations between maternal preeclampsia and several cardiovascular risk factors, including hypertension, obesity, and diabetes during childhood and early adulthood,^9,10,11,12^ are well established, knowledge regarding the association between preeclampsia and CVD risk in the offspring is limited. Some studies,^13,14,15,16^ but not all,^17,18^ found that maternal preeclampsia was associated with an increased risk of overall CVDs. However, due to the small sample size and short follow-up of these previous studies, most were unable to examine the associations of preeclampsia with specific CVDs, such as IHD and stroke, in the offspring.

Several other knowledge gaps remain. First, it is plausible that early- and late-onset preeclampsia have different outcomes given that they are 2 distinct entities,^19^ but few studies have investigated whether the risk of IHD and stroke differs according to the timing of onset or severity of preeclampsia. Second, preeclampsia has an association with fetal growth restriction (FGR)^9^ and preterm birth^20^; both factors, in turn, were found to be associated with increased CVD risks later in life.^21,22^ To our knowledge, no study has examined the role of FGR and preterm birth in the association between maternal preeclampsia and offspring risk of IHD and stroke. Third, it remains unclear whether such associations may be attributable to shared familial risk factors.

In this cohort study, we aimed to investigate (1) the association between maternal preeclampsia and risks of IHD and stroke in the offspring; (2) whether the association varies by severity or timing of onset of preeclampsia; and (3) the role of preterm birth and small for gestational age (SGA, a proxy for FGR) birth, both of which are related to preeclampsia and CVDs, in this association.

## Methods

### Study Population

We conducted this population-based cohort study by linking nationwide registers in Denmark, Finland, and Sweden using the unique personal identification number assigned to all residents of these countries (eTable 1 in the Supplement). The study was approved by the Danish Data Protection Agency and the Research Ethics Committee at Karolinska Institute in Stockholm. These boards do not request informed consent for register-based studies. We followed the Strengthening the Reporting of Observational Studies in Epidemiology (STROBE) reporting guideline.

Using the Medical Birth Registers, we included all live singleton births with a linkage to their mothers from 1973 to 2016 in Denmark and from 1973 to 2014 in Sweden, and 90% of live singleton births were randomly selected from 1987 to 2014 in Finland. We included only singleton births given concerns that register-based information on birth-related exposures and later outcomes may be mixed up within twin pairs.

### Measures

Information on preeclampsia was retrieved from the Danish National Patient Register,^23^ the Finnish Hospital Discharge Register,^24^ and the Swedish Medical Birth Registers, using the *International Classification of Diseases, Eighth Revision (ICD-8); International Classification of Diseases, Ninth Revision (ICD-9);* and/or *International Statistical Classification of Diseases and Related Health Problems, Tenth Revision (ICD-10)* diagnosis codes (eTable 2 in the Supplement). We also extracted information on preexisting chronic hypertension and gestational hypertension.

Using these *ICD* diagnosis codes, we classified preeclampsia into mild or moderate and severe (eTable 2 in the Supplement). Furthermore, we considered early-onset preeclampsia and SGA birth accompanying preeclampsia to be proxy markers of severity. In Denmark and Finland, we categorized early-onset and late-onset preeclampsia according to whether it was diagnosed before or after 34 weeks of gestation.^25^ Because date of diagnosis was not available in Sweden, we classified preeclampsia as early onset if delivery occurred before 34 completed weeks of gestation and as late onset if delivery occurred after 34 completed weeks of gestation. We defined SGA as a birth weight below the 10th percentile of the sex-specific and gestational age–specific standard curve for normal fetal growth.^26^

We retrieved information on IHD, stroke, and the 2 main subtypes of stroke (hemorrhagic stroke and ischemic stroke) from national patient and causes-of-death registers (eTable 3 in the Supplement). Follow-up started at birth and ended on the date of the first diagnosis of the outcome, death, emigration, or latest date with available data (December 31, 2016, in Denmark; December 31, 2014, in Finland and Sweden), whichever occurred first.

We obtained information on offspring characteristics (country of birth, calendar year of birth, sex, and diagnoses of congenital anomalies) and maternal characteristics (country of origin, educational level, marital status, age at delivery, parity, smoking status and body mass index [BMI; calculated as weight in kilograms divided by height in meters squared] during early pregnancy, diabetes status before delivery, and family history of CVDs) by linkage to several registers. Information on race and ethnicity was considered by law to be particularly sensitive and was not allowed to be collected; thus, we used country of origin as a proxy for ethnicity. A detailed description of the measurement of covariates and the criteria for confounder selection are presented in eAppendix 1 in the Supplement.

### Statistical Analysis

We used Cox proportional hazards regression models to estimate hazard ratios (HRs) and 95% CIs for IHD and stroke according to exposure, with attained age as the time scale. We examined the proportional hazards assumption using Schoenfeld residuals, but these suggested possible minor violations; therefore, we used flexible parametric survival models^27^ to visualize the time-varying associations. We also split the follow-up at age 18 years to investigate whether the associations differed between childhood and adulthood. We then analyzed whether the risks of IHD and stroke varied by the severity of preeclampsia and formally tested the differences. The associations of the other 2 types of hypertensive disorders of pregnancy (preexisting chronic hypertension and gestational hypertension) with IHD and stroke risks were estimated as well. In the main models, we adjusted for the available covariates for most study participants (offspring sex and calendar year of birth as well as maternal educational level, marital status, age at delivery, parity, and diabetes status before delivery); missing values were included as separate categories. Because information on maternal country of origin, smoking status and BMI in early pregnancy, and family history of CVDs was not available in all 3 countries or for the entire study period, we adjusted for these covariates in sensitivity analyses among participants with available data. To account for unmeasured familial confounders, we performed sibling analyses, as described in eAppendix 2 in the Supplement.

We did not adjust for SGA and preterm births in the main models because these outcomes may lie in the causal pathway between exposure and outcome. However, to explore their additional roles in the studied associations, we compared the individual and combined associations of preeclampsia and preterm and SGA births with the risk of IHD and stroke by creating a categorical variable with 8 levels according to preeclampsia (yes or no), preterm birth (yes or no), and SGA birth (yes or no). Furthermore, we performed formal tests of interaction to assess the interaction of preterm or SGA birth and preeclampsia with the risk of IHD and stroke. We also conducted stratified analyses and formal tests of interaction between preeclampsia and sex and country. We further examined the associations of IHD and stroke risks with preeclampsia among offspring without any congenital anomaly.

An association was considered to be statistically significant if *P* < .05. Statistical analyses were performed with SAS, version 9.4 (SAS Institute Inc); Stata, version 15.1 (StataCorp LLC); and RStudio, version 1.2.1578 (RStudio Inc). Data were analyzed between September 2020 and September 2022.

## Results

The cohort of 8 475 819 births (2 668 697 [31.5%] from Denmark, 1 636 116 [19.3%] from Finland, and 4 171 006 [49.2%] from Sweden) comprised 4 350 546 boys (51.3%) and 4 123 964 girls (48.7%). A total of 188 670 participants (2.2%) were exposed to maternal preeclampsia, 37 854 (0.5%) to preexisting chronic hypertension, and 104 595 (1.2%) to gestational hypertension. The prevalence of preeclampsia was 2.71% in Denmark, 2.04% in Sweden, and 1.91% in Finland. Among individuals with severe preeclampsia, 15 506 (33.3%) had early-onset preeclampsia and 19 056 (41.0%) had preeclampsia with SGA birth; among individuals with mild or moderate preeclampsia, 18 177 (13.3%) had early-onset preeclampsia and 26 456 (19.4%) had preeclampsia with SGA birth (eTable 4 in the Supplement).

During the median (IQR) follow-up of 19.3 (9.0-28.1) years, IHD and stroke were rare (ie, 7446 offspring [0.1%] were diagnosed with IHD, and 10 918 offspring [0.1%] were diagnosed with stroke). Compared with the offspring of individuals with normotensive pregnancy, the offspring of individuals with preeclampsia were more likely to have preterm birth and SGA birth, and individuals who delivered were more likely to be nulliparous, with obesity, single, a nonsmoker, and have a family history of CVDs (Table 1).

**Table 1.** Baseline Characteristics of the Study Population

Characteristic	No. (%)^a^			
	Preeclampsia (n = 188 670)	Gestational hypertension (n = 104 595)	Chronic hypertension (n = 37 854)	Normotensive pregnancy (n = 8 150 357)
Offspring characteristics				
Country of birth				
Denmark	72 259 (38.3)	25 884 (24.8)	12 990 (34.3)	2 561 557 (31.4)
Finland	31 265 (16.6)	44 883 (42.9)	15 782 (41.7)	1 545 850 (19.0)
Sweden	85 146 (45.1)	33 828 (32.3)	9 082 (24.0)	4 042 950 (49.6)
Calendar year of birth				
1973-1978	4 839 (2.6)	2 359 (2.3)	111 (0.3)	984 271 (12.1)
1979-1984	14 964 (7.9)	7 337 (7.0)	647 (1.7)	843 101 (10.3)
1985-1990	29 073 (15.4)	13 376 (12.8)	2 088 (5.5)	1 176 830 (14.4)
1991-1996	36 688 (19.5)	14 959 (14.3)	3 829 (10.1)	1 358 034 (16.7)
1997-2002	30 628 (16.2)	17 847 (17.0)	8 197 (21.7)	1 168 678 (14.4)
2003-2008	32 514 (17.2)	22 494 (21.5)	10 762 (28.4)	1 239 011 (15.2)
2009-2016	39 964 (21.2)	26 223 (25.1)	12 220 (32.3)	1 380 432 (16.9)
Sex				
Boy	97 912 (51.9)	55 088 (52.7)	19 368 (51.2)	4 180 198 (51.3)
Girl	90 731 (48.1)	49 496 (47.3)	18 468 (48.8)	3 967 887 (48.7)
Unknown	27 (0.01)	11 (0.01)	0	1 272 (0.02)
Any congenital anomaly				
No	165 504 (87.7)	94 340 (90.2)	33 618 (88.8)	7 413 915 (91.0)
Yes	23 166 (12.3)	10 255 (9.8)	4 236 (11.2)	736 442 (9.0)
Preterm birth (<37 gestational wk)				
No	146 684 (79.8)	96 845 (93.9)	33 622 (89.4)	7 380 747 (95.6)
Yes	37 199 (20.2)	6 245 (6.1)	4 236 (10.6)	338 544 (4.4)
SGA^b^				
No	137 359 (75.1)	86 258 (83.9)	31 352 (83.6)	6 960 459 (90.5)
Yes	45 512 (24.9)	16 532 (16.1)	6 127 (16.4)	734 328 (9.5)
Maternal characteristics				
Country of origin same as the country of birth^c^				
No	14 625 (9.3)	3 985 (6.7)	2 011 (9.1)	773 582 (11.7)
Yes	142 655 (90.6)	55 676 (93.2)	20 053 (90.8)	5 818 534 (88.1)
Unknown	125 (0.1)	51 (0.1)	8 (0.1)	12 391 (0.2)
Age at delivery, y				
≤19	6 005 (3.2)	2 016 (1.9)	159 (0.4)	240 677 (3.0)
20-24	38 019 (20.1)	16 066 (15.4)	2 362 (6.3)	1 588 783 (19.5)
25-29	63 596 (33.7)	33 059 (31.6)	8 433 (22.3)	2 870 923 (35.2)
30-34	50 121 (26.6)	31 014 (29.7)	13 036 (34.4)	2 322 255 (28.5)
≥35	30 929 (16.4)	22 440 (21.4)	13 864 (36.6)	1 127 677 (13.8)
Unknown	0	0	0	42 (<0.01)
Educational level before delivery				
Primary and lower secondary	33 476 (17.7)	10 782 (10.3)	3 406 (9.00)	1 457 757 (17.9)
Upper secondary	95 118 (50.4)	56 157 (53.7)	20 344 (53.7)	3 950 516 (48.5)
≥Bachelor’s degree	54 636 (29.0)	32 702 (31.3)	12 475 (33.0)	2 424 645 (29.7)
Unknown	5 440 (2.9)	4 954 (4.7)	1 629 (4.3)	317 439 (3.9)
Marital status before delivery				
Not married/registered partnership	99 690 (52.8)	48 206 (46.1)	16 176 (42.7)	3 530 821 (43.3)
Married/registered partnership	87 549 (46.4)	54 953 (52.5)	21 241 (56.1)	4 413 610 (54.2)
Unknown	1 431(0.8)	1 436 (1.4)	437 (1.2)	205 926 (2.5)
Parity				
1	124 795 (66.2)	63 862 (61.1)	16 444 (43.4)	3 779 841 (46.4)
2	40 078 (21.2)	23 222 (22.2)	11 473 (30.3)	2 747 272 (33.7)
≥3	23 648 (12.5)	17 415 (16.6)	9 917 (26.2)	1 618 740 (19.8)
Unknown	149 (0.1)	96 (0.1)	20 (0.1)	4 504 (0.1)
Smoking status during early pregnancy				
No	131 126 (69.5)	78 637 (75.2)	31 602 (83.5)	4 818 311 (59.1)
Yes	22 376 (11.9)	13 107 (12.5)	4 247 (11.2)	1 151 939 (14.1)
Unknown	35 168 (18.6)	12 851 (12.3)	2 005 (5.3)	2 180 107 (26.8)
BMI during early pregnancy^c^				
<18.5	1 928 (1.2)	665 (1.1)	280 (1.3)	126 702 (1.9)
18.5-24.9	40 931 (26.0)	16 660 (27.8)	6 417 (29.1)	1 966 797 (29.8)
25.0-29.9	25 931 (16.5)	10 640 (17.8)	4 236 (19.2)	740 266 (11.2)
≥30.0	19 221 (12.2)	7 946 (13.3)	4 274 (19.3)	303 743 (4.6)
Unknown	69 394 (44.1)	23 861 (40.0)	6 865 (31.1)	3 466 999 (52.5)
Diabetes status before delivery				
No	178 599 (94.7)	96 612 (92.4)	31 197 (82.4)	7 959 279 (97.7)
Yes	10 071 (5.3)	7 983 (7.6)	6 657 (17.6)	191 078 (2.3)
Family history of CVDs^c^				
No	115 739 (73.5)	41 675 (69.8)	12 300 (55.7)	5 221 193 (79.0)
Yes	41 666 (26.5)	18 037 (30.2)	9 772 (44.3)	1 383 314 (21.0)

Abbreviations: BMI, body mass index (calculated as weight in kilograms divided by height in meters squared); CVDs, cardiovascular diseases; SGA, small for gestational age.

^a^ Some individuals were exposed to more than 1 subtype of hypertension.

^b^ SGA was defined as birth weight below the 10th percentile of the sex-specific and gestational age–specific standard curve for normal fetal growth.

^c^ These covariates were only available in Sweden and Denmark.

### Preeclampsia and IHD Risk

We observed increased risk of IHD among the offspring of individuals with preeclampsia (adjusted HR, 1.33; 95% CI, 1.12-1.58), preexisting chronic hypertension (adjusted HR, 1.72; 95% CI, 1.02-2.92), or gestational hypertension (adjusted HR, 1.44; 95% CI, 1.12-1.84) compared with offspring of individuals with normotensive pregnancy (Table 2). The point estimates for IHD risk were slightly higher in case of preeclampsia with early onset or with SGA birth than preeclampsia with late onset or without SGA birth, but these differences were not statistically significant. In the sibling analyses, the risk estimates were generally attenuated, except for preeclampsia with early onset (adjusted HR, 2.58; 95% CI, 0.79-8.43) or SGA birth (adjusted HR, 1.55; 95% CI, 0.57-4.18) (Table 2).

**Table 2.** Incidence Rates and Hazard Ratios With 95% CIs for Ischemic Heart Disease According to Maternal Preeclampsia and Its Subtypes

Exposure	Population analysis (n = 8 475 819)				Sibling analysis (n = 7 031 134)			
	No. of events	Rate per 10 000 person-years	HR (95% CI)		No. of events	Rate per 10 000 person-years	HR (95% CI)	
			Crude	Adjusted^a^			Crude	Adjusted^a^
Normotensive pregnancy	7237	0.46	1 [Reference]	1 [Reference]	5 225	0.40	1 [Reference]	1 [Reference]
Preeclampsia	133	0.42	1.50 (1.26-1.78)	1.33 (1.12-1.58)	93	0.36	0.81 (0.58-1.12)	0.82 (0.59-1.15)
Onset of preeclampsia^b^								
Late onset	92	0.37	1.58 (1.28-1.94)	1.31 (1.06-1.61)	55	0.27	0.70 (0.45-1.10)	0.64 (0.37-1.11)
Early onset	19	0.41	2.08 (1.32-3.26)	1.62 (1.03-2.55)	13	0.38	1.60 (0.59-4.39)	2.58 (0.79-8.43)
Severity of preeclampsia^b^								
Mild or moderate	110	0.45	1.50 (1.24-1.81)	1.33 (1.10-1.61)	79	0.39	0.78 (0.55-1.11)	0.79 (0.55-1.13)
Severe	23	0.33	1.53 (1.01-2.30)	1.33 (0.89-2.01)	14	0.26	0.95 (0.42-2.13)	1.07 (0.47-2.45)
Preeclampsia with or without SGA^b^								
Without SGA	83	0.37	1.61 (1.30-2.01)	1.32 (1.06-1.64)	50	0.28	0.74 (0.47-1.17)	0.64 (0.36-1.13)
With SGA	28	0.39	1.78 (1.23-2.59)	1.50 (1.03-2.18)	18	0.33	1.04 (0.45-2.41)	1.55 (0.57-4.18)
Other hypertensive disorders during pregnancy								
Preexisting chronic hypertension	14	0.33	2.19 (1.30-3.70)	1.72 (1.02-2.92)	11	0.30	0.94 (0.34-2.63)	0.87 (0.30-2.50)
Gestational hypertension	64	0.41	1.56 (1.22-1.99)	1.44 (1.12-1.84)	54	0.42	0.89 (0.57-1.37)	0.91 (0.58-1.41)

Abbreviations: HR, hazard ratio; SGA, small for gestational age (birth weight below the 10th percentile of the sex-specific and gestational age–specific standard curve for normal fetal growth).

^a^ Analyses were adjusted for offspring calendar year of birth; offspring sex; and maternal parity, age, educational level, marital status, and diabetes status before childbirth.

^b^ The variables onset of preeclampsia (early vs late), severity of preeclampsia (mild or moderate vs severe), and preeclampsia with or without SGA were categorical variables, each with 3 subgroups including normotensive pregnancy as the reference for all 3 variables (1 for unexposed and 2 for exposed). The difference in the strength of the associations between less severe and severe forms of preeclampsia with ischemic heart disease risk was formally tested. The *P* values were *P* = .27 for the difference between late-onset and early-onset preeclampsia, *P* = .93 for the difference between mild or moderate and severe preeclampsia, and *P* = .64 for the difference between preeclampsia without SGA and with SGA.

**Figure 1.**
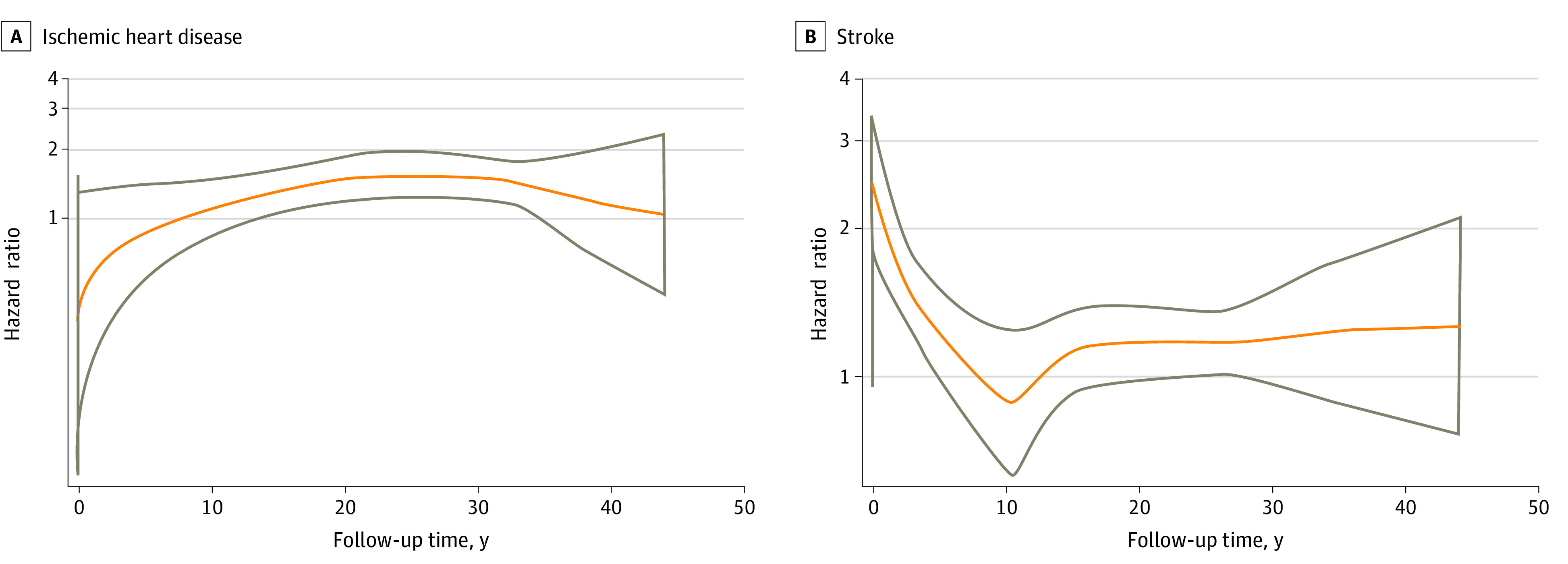
Adjusted Hazard Ratios (HRs) for Ischemic Heart Disease and Stroke From Flexible Parametric Survival Models Hazard ratios (orange lines) and 95% CIs (gray lines) for ischemic heart disease (A) and stroke (B) in offspring according to maternal preeclampsia. A spline with 5 degrees of freedom (4 intermediate knots and 2 knots at each boundary, placed at quintiles of the distribution of events) was used for the baseline rate, and a spline with 3 degrees of freedom was used for the time-varying effect. Analyses were adjusted for offspring calendar year of birth; offspring sex; and maternal parity, age, educational level, marital status, and diabetes status before childbirth.

The flexible parametric model and the cumulative risk curves showed no differences in IHD incidence rates by exposure in the first 18 years of follow-up. In later periods, however, offspring who were exposed had higher IHD risk than those who were unexposed to maternal preeclampsia (Figure 1; eFigure in the Supplement). A similar pattern was seen in the analysis when we split the follow-up at the age of 18 years, with lower IHD risk in the first 18 years of life (adjusted HR, 1.07; 95% CI, 0.73-1.57) than afterward (adjusted HR, 1.42; 95% CI, 1.20-2.07) (eTable 5 in the Supplement).

### Preeclampsia and Stroke Risk

Compared with the offspring who were unexposed, those who were exposed to maternal preeclampsia had higher risks of overall stroke (adjusted HR, 1.34; 95% CI, 1.17-1.52) as well as hemorrhagic stroke (adjusted HR, 1.23; 95% CI, 1.01-1.50) and ischemic stroke (adjusted HR, 1.44; 95% CI, 1.21-1.72) (Table 3; eTable 6 and eFigure in the Supplement). Severe forms of preeclampsia were associated with higher risk of stroke compared with less severe forms (severe vs mild or moderate: adjusted HR, 1.81 [95% CI, 1.41-2.32] vs 1.22 [95% CI, 1.05-1.42]; early vs late onset: adjusted HR, 2.55 [95% CI, 1.97-3.28] vs 1.18 [95% CI, 1.01-1.39]; with vs without SGA birth: adjusted HR, 1.84 [95% CI, 1.44-2.34] vs 1.25 [95% CI, 1.07-1.48]). Offspring born after the other types of hypertensive disorders during pregnancy also had increased risk of stroke. In the sibling analyses, the adjusted HR for preeclampsia was attenuated, but the HRs for severe preeclampsia or preeclampsia with early onset or SGA birth were consistently higher than the less severe forms (severe vs mild or moderate: adjusted HR, 1.78 [95% CI, 1.10-2.90] vs 0.99 [95% CI, 0.77-1.30]; early vs late onset: adjusted HR, 1.81 [95% CI, 0.99-3.33] vs 1.11 [95% CI, 0.80-1.54]; with vs without SGA birth: adjusted HR, 2.10 [95% CI, 1.20-3.69] vs 1.06 [95% CI, 0.76-1.48]).

**Table 3.** Incidence Rates and Hazard Ratios With 95% CIs for Stroke According to Maternal Preeclampsia and Its Subtypes

Exposure	Population analysis (n = 8 475 819)				Sibling analysis (n = 7 031 134)			
	No. of events	Rate per 10 000 person-years	HR (95% CI)		No. of events	Rate per 10 000 person-years	HR (95% CI)	
			Crude	Adjusted^a^			Crude	Adjusted^a^
Normotensive pregnancy	10 546	0.66	1 [Reference]	1 [Reference]	7996	0.61	1 [Reference]	1 [Reference]
Preeclampsia	233	0.74	1.42 (1.25-1.62)	1.34 (1.17-1.52)	183	0.72	1.13 (0.89-1.43)	1.12 (0.88-1.42)
Onset of preeclampsia^b^								
Late onset	156	0.62	1.28 (1.09-1.50)	1.18 (1.01-1.39)	112	0.56	1.07 (0.80-1.44)	1.11 (0.80-1.54)
Early onset	60	1.28	2.88 (2.23-3.71)	2.55 (1.97-3.28)	44	1.28	1.66 (0.93-2.94)	1.81 (0.99-3.33)
Severity of preeclampsia^b^								
Mild or moderate	171	0.70	1.29 (1.11-1.50)	1.22 (1.05-1.42)	131	0.65	1.02 (0.78-1.33)	0.99 (0.77-1.30)
Severe	62	0.89	1.94 (1.51-2.49)	1.81 (1.41-2.32)	52	0.95	1.77 (1.09-2.87)	1.78 (1.10-2.90)
Preeclampsia with or without SGA^b^								
Without SGA	148	0.66	1.37 (1.16-1.61)	1.25 (1.07-1.48)	104	0.58	1.03 (0.76-1.39)	1.06 (0.76-1.48)
With SGA	67	0.94	1.99 (1.57-2.54)	1.84 (1.44-2.34)	52	0.96	1.81 (1.07-3.05)	2.10 (1.20-3.69)
Other hypertensive disorders during pregnancy								
Preexisting chronic hypertension	30	0.70	1.80 (1.25-2.57)	1.60 (1.12-2.30)	25	0.69	1.96 (1.01-3.82)	1.99 (1.01-3.90)
Gestational hypertension	115	0.74	1.46 (1.21-1.75)	1.40 (1.16-1.68)	93	0.73	1.43 (1.02-2.00)	1.45 (1.03-2.02)

Abbreviations: HR, hazard ratio; SGA, small for gestational age (birth weight below the 10th percentile of the sex-specific and gestational age–specific standard curve for normal fetal growth).

^a^ Analyses were adjusted for offspring calendar year of birth; offspring sex; and maternal parity, age, educational level, marital status, and diabetes status before childbirth.

^b^ The variables onset of preeclampsia (early vs late), severity of preeclampsia (mild or moderate vs severe), and preeclampsia with or without SGA were categorical variables, each with 3 subgroups including normotensive pregnancy as the reference for all 3 variables (1 for unexposed and 2 for exposed). The difference in the strength of the associations between less severe and severe forms of preeclampsia with stroke risk was formally tested. The *P* values were *P* < .001 for the difference between late-onset and early-onset preeclampsia, *P* = .006 for the difference between mild or moderate and severe preeclampsia, and *P* = .01 for the difference between preeclampsia without SGA and with SGA.

The flexible parametric model showed that the stroke risk associated with preeclampsia was highest in infancy, followed by a rapid decline until the age of 10 years. After age 18 years, the magnitude of the association remained relatively constant (Figure 1). The stroke risk associated with preeclampsia was higher in childhood than in adulthood (eTable 5 in the Supplement).

### Additional Role of Preterm and SGA Births

**Figure 2.**
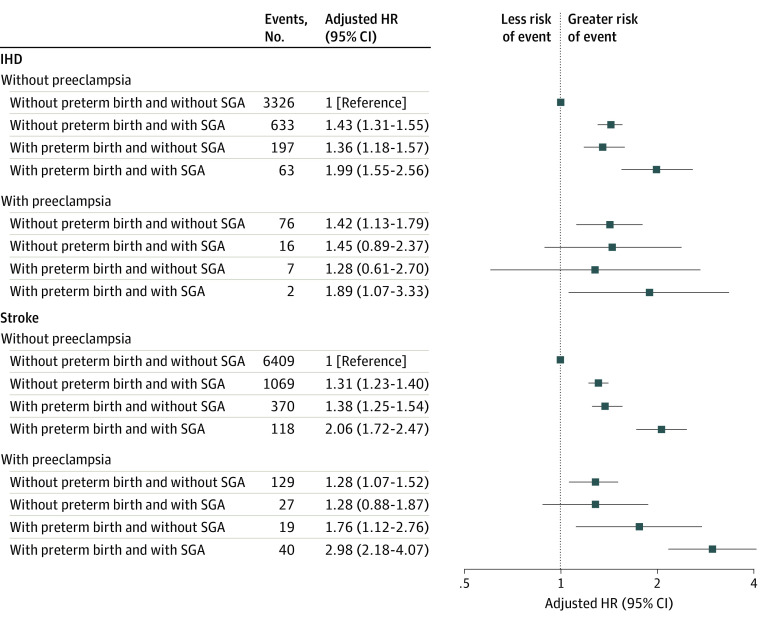
Hazard Ratios (HRs) for Ischemic Heart Disease (IHD) and Stroke According to Preeclampsia, Preterm Birth, and Small for Gestational Age (SGA) Offspring born to individuals without any hypertensive disorders of pregnancy were the reference, and analyses were adjusted for offspring calendar year of birth; offspring sex; and maternal parity, age, educational level, marital status, and diabetes status before childbirth. In case of IHD, the *P* values for interaction were *P* = .27 between preeclampsia and SGA and *P* = .31 between preeclampsia and preterm birth. In case of stroke, the *P* values for interaction were *P* = .56 between preeclampsia and SGA and *P* = .11 between preeclampsia and preterm birth.

There were no significant interactions between preeclampsia and SGA or preterm birth for IHD and stroke risks (Figure 2). The associations between preeclampsia and IHD (adjusted HR, 1.42; 95% CI, 1.13-1.79) and stroke (adjusted HR, 1.28; 95% CI, 1.07-1.52) risks persisted among individuals without preterm or SGA birth. Individuals with all 3 risk factors had 89% higher risk of IHD and 198% higher risk of stroke than individuals without any risk factor.

### Sensitivity Analyses

The associations between preeclampsia and IHD and stroke risks did not differ by country of birth or sex (eTables 7 and 8 in the Supplement). The results did not substantially change when we (1) restricted the analyses to offspring without any congenital anomaly or (2) adjusted for maternal country of origin, smoking status or BMI during early pregnancy, or family history of CVDs in addition to factors in the main model (eTables 9-13 in the Supplement).

## Discussion

This cohort study found that maternal preeclampsia and other hypertensive disorders of pregnancy were associated with increased risks of IHD and stroke in the offspring. The associations between preeclampsia and IHD and stroke risks were not fully explained by SGA and preterm births. The risk of stroke was higher for severe or early-onset preeclampsia or preeclampsia with SGA birth than for milder preeclampsia; these associations were observed also in the sibling analyses.

Results from the few studies in this area have been inconsistent. A study conducted in Helsinki, Finland, found that individuals born from 1934 to 1944 who were exposed prenatally to preeclampsia or gestational hypertension had an increased risk of stroke^16^ but not of IHD. The Northern Finland 1966 Birth Cohort Study found no association between maternal hypertension during pregnancy and the risk of stroke in the offspring but had limited power to assess specific associations with preeclampsia.^17^ A Danish study that followed up participants to age 27 years^18^ found no association between preeclampsia and risk of overall CVDs, possibly due to the low prevalence of CVDs in young adults. In contrast, other studies conducted in the US^14^ and Israel^15^ reported that maternal hypertensive disorders during pregnancy were associated with an increased risk of overall CVDs in offspring. Nevertheless, these studies had limited statistical power to investigate the association with specific heart or cerebrovascular diseases.

Moreover, few studies have evaluated the associations with specific subtypes of preeclampsia, which may lead to CVDs through different mechanisms. A recent study that used data from a cohort largely overlapping with the Danish subcohort of the present study^13^ found that the highest risk of overall CVDs was observed in cases of severe or early-onset preeclampsia. However, the lack of power limited the possibility to conduct a thorough examination of the associations of preeclampsia and its subtypes with IHD or stroke risk in young adults.

We believe this large cohort study extends the evidence in this field in several aspects. The higher stroke risk associated with severe rather than mild preeclampsia suggested a dose-response association. In line with recommendations from international guidelines,^28^ we also considered maternal preeclampsia accompanied by SGA birth to be severe preeclampsia and found it to be associated with greater risk of stroke than preeclampsia without SGA birth. Furthermore, we differentiated between early- and late-onset preeclampsia, as they have been shown to have different pathogenesis, biomarkers, and roles in the offspring’s cardiovascular risk profile.^29^ The associated risks were greater in early-onset preeclampsia than late-onset preeclampsia, reinforcing the possibility of 2 distinct entities. The comparable associations for hemorrhagic and ischemic stroke observed in this study suggested that maternal preeclampsia was a risk factor for both types of stroke. There was a pattern of similar associations for IHD, but the results were not conclusive. We also observed associations between preeclampsia and IHD and stroke risks among offspring without preterm or SGA birth, which suggested that preeclampsia was associated with increased CVD risk through pathways other than prematurity and FGR.

There are 2 potential explanations for the association between maternal preeclampsia and increased IHD and stroke risks: (1) shared familial environmental and genetic factors and (2) developmental programming. The sibling analyses yielded generally lower risks than the cohort analyses, suggesting that shared genetic factors or adverse environmental conditions^30^ may contribute to the observed associations. Nevertheless, in the sibling analyses, we observed a modestly increased risk of stroke and a pattern in this direction for IHD risk among offspring who were exposed to severe forms of preeclampsia compared with those who were exposed to normotensive pregnancy, suggesting that preeclampsia, particularly its severe forms, was associated with increased risk of CVDs given its role in fetal programming. The preeclamptic intrauterine environment is characterized by a variety of insults, such as placental insufficiency, endothelial dysfunction, hypoxia, raised antiangiogenic factors and oxidative stress, and inflammation.^31^ These in utero insults have various implications for the fetus, including changes in the renin-angiotensin-aldosterone and the immune system that lead to endothelial and cardiac dysfunction, abnormal vascular structure and cardiac remodeling, and accelerated atherosclerosis progression, which in turn have been associated with increased risk of IHD or stroke.^30^ Mild preeclampsia often occurs in women with cardiometabolic diseases,^32^ and thus its association with CVDs may be more likely due to familial confounding. In contrast, severe preeclampsia is more likely to be a placentation disorder characterized by placental vascular lesions and reduced placental perfusion^32^ and may have a CVD outcome through a process related to fetal programming.

### Limitations

Some limitations of this study need to be noted. First, the findings apply only to childhood and young adulthood because we could not examine the risks of IHD and stroke in middle age or older age. Second, some misclassification of exposure is likely given that the clinical definition of preeclampsia has varied among countries and over the years, and some cases of mild preeclampsia may not have been identified in the registers. However, validation studies have shown that preeclampsia in the registers has a high positive predictive value (Danish National Patient Register: 74%; Swedish Medical Birth Registers: 96%).^33,34^ Because the registration of exposure occurred before the outcomes, any misclassification of preeclampsia is expected to be nondifferential and to attenuate the observed associations. Similarly, although for thoroughness we analyzed associations with chronic hypertension and gestational hypertension, we did not investigate them in more depth given that the validity of these 2 hypertensive disorders in the Nordic registers was modest.^34^

Third, we tried to address residual confounding using sibling analysis, but this design also has some limitations. Sibling design assumes an absence of carryover effects between pregnancies (ie, the conditions of the first pregnancy should not change the exposure-outcome association in subsequent pregnancies). Any violation of this assumption may bias the estimates toward null.^35^ In addition, the sibling comparison design can amplify confounding by unmeasured confounders that are not shared by sibling pairs and may control for mediators that are shared by siblings.^36^ Fourth, statistical power in some of the subanalyses was likely limited. For example, our finding of a pattern toward a reversed outcome in the association between milder forms of preeclampsia and IHD risk in the sibling analyses may attract speculations of whether the outcome was by chance or implied that the exposed sibling, for whom preeclampsia did not affect the growth of or trigger an early-onset delivery, might in some way be more resilient than the unexposed sibling. We hope that future studies that are better powered may explore these questions further. Similarly, the number of individuals with preeclampsia superimposed on chronic hypertension was too limited to allow sufficiently powered analyses. Fifth, the results may be generalized primarily to singleton births in countries that have sociocultural contexts and health care systems similar to those of the Nordic countries in this study.

## Conclusions

This cohort study found that maternal preeclampsia during pregnancy was associated with increased risks of IHD and stroke in the offspring up to early adulthood and that the associated risks in case of stroke were higher for severe than for mild or moderate forms of preeclampsia. These associations were not fully explained by preterm or SGA birth. If these findings are confirmed by future studies, screening for risk factors among offspring born to individuals with preeclampsia and primary preventive measures may be implemented early in life to reduce the burden of CVDs.

## Notes

Supplement. **eAppendix 1.** Description of the Source, the Measurement, and the Categorization of Covariates **eAppendix 2.** Description of Our Sibling Analysis **eTable 1.** Description of Danish, Finnish, and Swedish Registers Used in This Study **eTable 2.** International Classification of Diseases Codes for Hypertensive Disorders of Pregnancy **eTable 3**. International Classification of Diseases Codes for Ischemic Heart Disease and Stroke **eTable 4.** The Number and Rates of Preeclampsia According to Its Severity **eTable 5**. Incidence Rates and Hazard Ratios With 95% Confidence Intervals for Ischemic Heart Disease and Stroke According to Preeclampsia, by Attained Age **eTable 6**. Incidence Rates and Hazard Ratios With 95% Confidence Intervals for Stroke Subtypes According to Maternal Preeclampsia and Its Subtypes **eTable 7**. Incidence Rates and Hazard Ratios With 95% Confidence Intervals for Ischemic Heart Disease and Stroke According to Preeclampsia, Stratified by Study Country **eTable 8**. Incidence Rates and Hazard Ratios With 95% Confidence Intervals for Ischemic Heart Disease and Stroke According to Preeclampsia, Stratified by the Child’s Sex **eTable 9.** Incidence Rates and Hazard Ratios With 95% Confidence Intervals for Ischemic Heart Disease and Stroke According to Preeclampsia and Its Subtypes, in Children Without Congenital Anomalies **eTable 10.** Incidence Rates and Hazard Ratios With 95% Confidence Intervals for Ischemic Heart Disease and Stroke According to Preeclampsia and Its Subtypes, in Children With Information on Maternal Country of Origin **eTable 11.** Incidence Rates and Hazard Ratios With 95% Confidence Intervals for Ischemic Heart Disease and Stroke According to Preeclampsia and Its Subtypes, in Children With Information on Maternal Smoking During Early Pregnancy **eTable 12.** Incidence Rates and Hazard Ratios With 95% Confidence Intervals for Ischemic Heart Disease and Stroke According to Preeclampsia and Its Subtypes, in Children With Information on Maternal Body Mass Index During Early Pregnancy **eTable 13.** Incidence Rates and Hazard Ratios With 95% Confidence Intervals for Ischemic Heart Disease and Stroke According to Preeclampsia and Its Subtypes, in Children With Information on Maternal Family History of Cardiovascular Diseases **eFigure.** Cumulative Incidence of A, Ischemic Heart Disease, and B, Stroke According to Preeclampsia
