# Supplementary material for: Association of Maternal Preeclampsia With Offspring Risks of Ischemic Heart Disease and Stroke in Nordic Countries
Source: JAMA Netw Open. 2022 Nov 15;5(11):e2242064. doi: 10.1001/jamanetworkopen.2022.42064 (PMC9667328; doi:10.1001/jamanetworkopen.2022.42064)
Supplement: Supplement. — eAppendix 1. Description of the Source, the Measurement, and the Categorization of Covariates eAppendix 2. Description of Our Sibling Analysis eTable 1. Description of Danish, Finnish, and Swedish Registers Used in This Study eTable 2. International Classification of Diseases Codes for Hypertensive Disorders of Pregnancy eTable 3. International Classification of Diseases Codes for Ischemic Heart Disease and Stroke eTable 4. The Number and Rates of Preeclampsia According to Its Severity eTable 5. Incidence Rates and Hazard Ratios With 95% Confidence Intervals for Ischemic Heart Disease and Stroke According to Preeclampsia, by Attained Age eTable 6. Incidence Rates and Hazard Ratios With 95% Confidence Intervals for Stroke Subtypes According to Maternal Preeclampsia and Its Subtypes eTable 7. Incidence Rates and Hazard Ratios With 95% Confidence Intervals for Ischemic Heart Disease and Stroke According to Preeclampsia, Stratified by Study Country eTable 8. Incidence Rates and Hazard Ratios With 95% Confidence Intervals for Ischemic Heart Disease and Stroke According to Preeclampsia, Stratified by the Child’s Sex eTable 9. Incidence Rates and Hazard Ratios With 95% Confidence Intervals for Ischemic Heart Disease and Stroke According to Preeclampsia and Its Subtypes, in Children Without Congenital Anomalies eTable 10. Incidence Rates and Hazard Ratios With 95% Confidence Intervals for Ischemic Heart Disease and Stroke According to Preeclampsia and Its Subtypes, in Children With Information on Maternal Country of Origin eTable 11. Incidence Rates and Hazard Ratios With 95% Confidence Intervals for Ischemic Heart Disease and Stroke According to Preeclampsia and Its Subtypes, in Children With Information on Maternal Smoking During Early Pregnancy eTable 12. Incidence Rates and Hazard Ratios With 95% Confidence Intervals for Ischemic Heart Disease and Stroke According to Preeclampsia and Its Subtypes, in Children With Information on Maternal Body Mass Index During Early Pre [file jamanetwopen-e2242064-s001.pdf]

Supplemental Online Content

Yang F, Janszky I, Gissler M, et al. Association of maternal preeclampsia with offspring risks of ischemic heart disease and stroke in Nordic countries. *JAMA Netw Open*. 2022;5(11):e2242064. doi:10.1001/jamanetworkopen.2022.42064

eAppendix 1. Description of the Source, the Measurement, and the Categorization of Covariates
eAppendix 2. Description of Our Sibling Analysis
eTable 1. Description of Danish, Finnish, and Swedish Registers Used in This Study
eTable 2. International Classification of Diseases Codes for Hypertensive Disorders of Pregnancy
eTable 3. International Classification of Diseases Codes for Ischemic Heart Disease and Stroke
eTable 4. The Number and Rates of Preeclampsia According to Its Severity
eTable 5. Incidence Rates and Hazard Ratios With 95% Confidence Intervals for Ischemic Heart Disease and Stroke According to Preeclampsia, by Attained Age
eTable 6. Incidence Rates and Hazard Ratios With 95% Confidence Intervals for Stroke Subtypes According to Maternal Preeclampsia and Its Subtypes
eTable 7. Incidence Rates and Hazard Ratios With 95% Confidence Intervals for Ischemic Heart Disease and Stroke According to Preeclampsia, Stratified by Study Country
eTable 8. Incidence Rates and Hazard Ratios With 95% Confidence Intervals for Ischemic Heart Disease and Stroke According to Preeclampsia, Stratified by the Child's Sex
eTable 9. Incidence Rates and Hazard Ratios With 95% Confidence Intervals for Ischemic Heart Disease and Stroke According to Preeclampsia and Its Subtypes, in Children Without Congenital Anomalies
eTable 10. Incidence Rates and Hazard Ratios With 95% Confidence Intervals for Ischemic Heart Disease and Stroke According to Preeclampsia and Its Subtypes, in Children With Information on Maternal Country of Origin
eTable 11. Incidence Rates and Hazard Ratios With 95% Confidence Intervals for Ischemic Heart Disease and Stroke According to Preeclampsia and Its Subtypes, in Children With Information on Maternal Smoking During Early Pregnancy
eTable 12. Incidence Rates and Hazard Ratios With 95% Confidence Intervals for Ischemic Heart Disease and Stroke According to Preeclampsia and Its Subtypes, in Children With Information on Maternal Body Mass Index During Early Pregnancy
eTable 13. Incidence Rates and Hazard Ratios With 95% Confidence Intervals for Ischemic Heart Disease and Stroke According to Preeclampsia and Its Subtypes, in Children With Information on Maternal Family History of Cardiovascular Diseases
eFigure. Cumulative Incidence of A, Ischemic Heart Disease, and B, Stroke According to Preeclampsia

© 2022 Yang F et al. *JAMA Network Open.*

This supplemental material has been provided by the authors to give readers additional information about their work.

© 2022 Yang F et al. *JAMA Network Open.*

eAppendix 1. Description of the source, the measurement, and the categorization of covariates

Information on calendar year of birth (1973-1978, 1979-1984, 1985-1990, 1991-1996, 1997-2002, 2003-2008, 2009-2016), sex (boy or girl), gestational age, birth weight, and multiple/singleton birth was obtained from the Medical Birth Registers (MBR) in Denmark, Finland, and Sweden. Gestational age was based on routine ultrasound examinations performed in early second trimester or date of last menstrual period (LMP). Ultrasound examinations was not uniformly introduced until the mid-1990s in Denmark, Sweden and Finland. In the rare event of missing information on ultrasound examinations, we used LMP (if it was available) to estimate gestational age. We excluded offspring with abnormal gestational age (<154 or >315 days) or with abnormal birth weight (more than five standard deviations below or above the mean).

Diagnoses of congenital anomalies in study participants were extracted from the Danish National Patient Register (DNPR), the Swedish Patient Register (SPR) and the Finnish Hospital Discharge Register (FHDR) using the International Statistical Classification of Diseases and Related Health Problems (ICD) codes: ICD-8/9: 740-759 and ICD-10: Q00-Q99.

Maternal country of origin (same as the study country versus not) and marital status before delivery (married/registered partnership versus not) was identified from the Danish Civil Registration System, the Finnish Central Population Register, and the Swedish Total Population Register. Data on the mother's highest education before delivery was obtained from the Danish Integrated Database for Labour Market Research, the Education Register at Statistics Finland, and the Swedish Register of Education. From the MBR we also obtained data on maternal age at delivery (≤19, 20-24, 25-29, 30-34, and ≥35 years), parity (1,2, ≥3), body mass index (BMI, not available in our Finnish data) and smoking (yes or no) in early pregnancy. Maternal BMI was categorized as underweight (BMI <18.5 kg/m^2), normal weight (18.5–24.9 kg/m^2), overweight (25.0–29.9 kg/m^2), and obesity (≥30.0 kg/m^2).

Information on maternal diabetes before delivery (including pregestational diabetes and gestational diabetes mellitus) was retrieved from the DNPR, FHDR, and Swedish MBR using the following ICD codes: (1) in Denmark: ICD-8: 249, 250; ICD-10: E10-E14, O24; (2) in Finland: ICD-9: 250, 648A, 6488A; ICD-10: E10-E14, O24; and (3) in Sweden: ICD-8: 250; ICD-9: 250, 648A, 648W; ICD-10: E10-E14, O24. To retrieve information on maternal family history of CVDs before delivery, we identified the mother's family members from the Danish Civil Registration System and the Swedish Multi-Generation Register, and then extracted their CVD diagnoses from the DNPR, the Danish Register of Cause of Death, the SPR, and the Swedish Cause of Death Register using ICD-8/9: 390–459 and ICD-10: I00-I99.

Our criteria for selecting confounders were (1) a known or an a-priori hypothesized association with both exposure and outcome and (2) not being on the causal pathway between exposure and outcome.

eAppendix 2. Description of our sibling analysis

The sibling analysis included all sibling pairs from our whole population. Firstly, we identified the mothers who had two or more biological children, and created a sub-cohort of these sibling pairs. Secondly, we performed analyses in this sibling sub-cohort using a stratified Cox regression with a separate stratum for each family identified by the mother's identification number. We adjusted for the same covariates as in the analyses involving the whole population. Only sibling pairs discordant for exposure and outcome contributed to the estimates in the analyses.

The sibling analysis included 7 031 134 sibling pairs with 2 808 293 mothers, among them 15 851 mothers had recurrent preeclampsia. The number of sibling pairs discordant for exposure to maternal preeclampsia and the diagnosis of ischemic heart disease or stroke in their lives was 290 027, 14 093, or 21 998, respectively.

© 2022 Yang F et al. *JAMA Network Open.*

eTable 1. Description of Danish, Finnish, and Swedish registers used in this study

Data source	Information	Period covered
**Denmark**		
Danish Civil Registration System	Sex, birth date, place of birth, vital status, linkage to parents and siblings, marital status, and migration	1968-2016
Danish Integrated Database for Labour Market Research	Mother's highest completed educational attainment	1980-2016
Danish Medical Birth Register	Child's birth date, sex, gestational age, birth weight, singleton status, mother's body mass index (available since 2003) and smoking in early pregnancy (available since 1991), age at delivery and parity	1973-2016
Danish National Patient Register	Information on inpatient and outpatient care (diagnosis, date)	Inpatient care: 1977-2016; outpatient care and emergency department contacts: 1995-2016
Danish Register of Causes of Death	Date of death and cause of death	1970-2016
**Finland**		
Finnish Central Population Register	Sex, date of birth, death, marital status, and migration	1968-2014
Education Register at Statistics Finland	Mother's highest completed educational attainment	1970-2014
Finnish Medical Birth Register	Child's birth date, sex, gestational age, birth weight, singleton status, mother's smoking in early pregnancy, age at delivery and parity	1987-2014
Finnish Hospital Discharge Register	Information on inpatient and outpatient care (diagnosis, date)	Inpatient care: 1969-2014; outpatient care contacts: 1998-2014
Finnish Causes of Death Register	Date of death and cause of death	1969-2014
**Sweden**		
Swedish Total Population Register	Sex, birth date, place of birth, civil status, marital status, and migration	1968-2014

© 2022 Yang F et al. *JAMA Network Open.*

Swedish Multi-Generation Register	Relationships for all residents born since 1932. The index persons must have been registered since 1961 in order to be included.	1961-2014
Swedish Register of Education	Mother's highest completed educational attainment	1985-2014
Swedish Medical Birth Register	Child's birth date, sex, gestational age, birth weight, singleton status, mother's age at delivery, parity, complications during pregnancy, smoking and body mass index during pregnancy (available since 1982)	1973-2014
Swedish Patient Register	Information on inpatient and outpatient care (diagnosis, date)	Inpatient care: 1964-2014 (its coverage became nationwide since 1987); outpatient care: 2001-2014
Swedish Cause of Death Register	Date and cause of death	1952-2014

© 2022 Yang F et al. *JAMA Network Open.*

eTable 2. International Classification of Diseases codes for hypertensive disorders of pregnancy

	ICD-8	ICD-9	ICD-10
**Pre-existing chronic hypertension**			
Denmark [a]	400.09, 400.19, 400.29, 400.39, 400.99, 401.99		I10, I11, I12, I13, I15, O10, O11
Finland [b]		4019, 4029, 4039, 4040, 4059, 6420, 6421, 6422, 6427	I10, I11, I12, I13, I15, O10, O11
Sweden [c]	400, 401, 402, 403, 404	401, 402, 403, 404, 405, 642A, 642B, 642C, 642H	I10, I11, I12, I13, I15, O10, O11
**Gestational hypertension**			
Denmark [a]	637.00, 760.29		O13, O16
Finland [b]		6423, 6429	O13, O16
Sweden [c]	637.01	642D, 642X	O13, O16
**Preeclampsia**			
Denmark [a]	637.03, 637.04, 637.09, 637.19		O14, O15
Finland [b]		6424, 6425, 6426	O14, O15
Sweden [c]	637.03, 637.04, 637.09, 637.99, 637.10	642E, 642F, 642G	O14, O15
**Mild/moderate preeclampsia**			
Denmark [a]	637.03, 637.09		O14.0, O14.9
Finland [b]		6424	O14.0, O14.9
Sweden [c]	637.03, 637.09, 637.99	642E	O14.0, O14.9
**Severe preeclampsia**			
Denmark [a]	637.04, 637.19		O14.1, O14.2, O15
Finland [b]		6425, 6426	O14.1, O15
Sweden [c]	637.04, 637.10	642F, 642G	O14.1, O14.2, O15

Abbreviations: ICD, International Statistical Classification of Diseases and Related Health Problems.

[a] In Denmark, the information about maternal hypertensive disorders during pregnancy was extracted from the Danish National Patient Register. We used ICD-8 up to 1995 and ICD-10 afterwards.

[b] In Finland, the information about maternal hypertensive disorders during pregnancy was extracted from the Finnish Hospital Discharge Register. We used of ICD-9 up to 1995 and ICD-10 afterwards.

[c] In Sweden, the information about maternal hypertensive disorders during pregnancy was extracted from the Swedish Medical Birth Register. We used ICD-8 up to 1986, ICD-9 from 1987 to 1996, and ICD-10 afterwards.

© 2022 Yang F et al. *JAMA Network Open.*

eTable 3. International Classification of Diseases codes for ischemic heart disease and stroke

	ICD-8	ICD-9	ICD-10
**Ischemic heart disease**			
Denmark [a]	410-414		I20-I25
Finland [b]		4100, 4109, 4110, 4120, 4121, 4131, 4140, 4148, 4149	I20-I25
Sweden [c]	410-414	410-414	I20-I25
**Stroke**			
Denmark [a]	430, 431, 433, 434, 436		I60, I61, I63, I64
Finland [b]		430, 431, 433, 434, 436	I60, I61, I63, I64
Sweden [c]	430, 431, 433, 434, 436	430, 431, 433, 434, 436	I60, I61, I63, I64
**Hemorrhagic stroke**			
Denmark [a]	430, 431		I60, I61
Finland [b]		430, 431	I60, I61
Sweden [c]	430, 431	430, 431	I60, I61
**Ischemic stroke**			
Denmark [a]	433, 434		I63, I64
Finland [b]		433, 434	I63, I64
Sweden [c]	433, 434	433, 434	I63, I64

Abbreviations: ICD, International Statistical Classification of Diseases and Related Health Problems.

[a] In Denmark, the information about ischemic heart disease and stroke and its subtypes was extracted from the Danish National Patient Register and the Danish Register of Cause of Death. We used the Danish version of ICD-8 up to 1995 and ICD-10 afterwards.

[b] In Finland, the information about ischemic heart disease and stroke and its subtypes was extracted from the Finnish Hospital Discharge Register and Finnish Caused of Death Register. We used the Finnish version of ICD-9 up to 1995 and ICD-10 afterwards.

[c] In Sweden, the information about ischemic heart disease and stroke and its subtypes was extracted from the Swedish Patient Register and the Swedish Cause of Death Register. We used the Swedish version of ICD-8 up to 1986, ICD-9 from 1987 to 1996, and ICD-10 afterwards.

© 2022 Yang F et al. *JAMA Network Open.*

eTable 4. The number and rates of preeclampsia according to its severity

	**Mild/moderate preeclampsia N (%)**	**Severe preeclampsia N (%)**
**Timing of onset**		
Late-onset	118 187 (86.7)	31 001 (66.7)
Early-onset	18 177 (13.3)	15 506 (33.3)
**Preeclampsia with/without SGA**		
Without SGA	109 908 (80.6)	27 451 (59.0)
With SGA	26 456 (19.4)	19 056 (41.0)

Abbreviations: SGA, small for gestational age (i.e., birth weight below the 10th percentile of the Swedish sex-specific standard curve for normal fetal growth); HR

© 2022 Yang F et al. *JAMA Network Open.*

eTable 5. Incidence rates and hazard ratios with 95% confidence intervals for ischemic heart disease and stroke according to preeclampsia, by attained age

Exposure	Number of events	Rate, per 10,000 person-years	Crude HR (95% CI)	Adjusted HR (95% CI) [a]
**Ischemic heart disease**				
**Attained age <18 years**				
Normotensive pregnancy	1,021	0.09	1.00	1.00
Preeclampsia	27	0.11	1.23 (0.84-1.80)	1.07 (0.73-1.57)
**Attained age ≥18 years**				
Normotensive pregnancy	6,216	1.30	1.00	1.00
Preeclampsia	106	1.45	1.59 (1.31-1.93)	1.42 (1.20-2.07)
**Stroke**				
**Attained age <18 years**				
Normotensive pregnancy	3,408	0.31	1.00	1.00
Preeclampsia	126	0.52	1.69 (1.41-2.02)	1.57 (1.31-1.88)
**Attained age ≥18 years**				
Normotensive pregnancy	7,137	1.49	1.00	1.00
Preeclampsia	107	1.47	1.20 (0.99-1.45)	1.13 (0.93-1.37)

Abbreviations: HR, hazard ratio; CI, confidence interval.

[a] We adjusted for calendar year of birth, sex, maternal parity, age, education, marital status, and diabetes before childbirth.

© 2022 Yang F et al. *JAMA Network Open.*

eTable 6. Incidence rates and hazard ratios with 95% confidence intervals for stroke subtypes according to maternal preeclampsia and its subtypes

Exposure	Hemorrhagic stroke				Ischemic stroke			
	Number of events	Rate, per 10,000 person-years	Crude HR (95% CI)	Adjusted HR (95% CI) [a]	Number of events	Rate, per 10,000 person-years	Crude HR (95% CI)	Adjusted HR (95% CI) [a]
**Normotensive pregnancy**	4,848	0.31	1.00	1.00	5,597	0.35	1.00	1.00
**Preeclampsia**	101	0.32	1.25 (1.03-1.53)	1.23 (1.01-1.50)	131	0.42	1.61 (1.35-1.91)	1.44 (1.21-1.72)
**Onset of preeclampsia**								
Late onset	65	0.26	1.07 (0.84-1.37)	1.03 (0.81-1.32)	91	0.36	1.51 (1.23-1.86)	1.34 (1.09-1.65)
Early onset	27	0.58	2.61 (1.79-3.82)	2.50 (1.71-3.66)	32	0.68	3.13 (2.21-4.43)	2.55 (1.80-3.61)
**Severity of preeclampsia**								
Mild/moderate	73	0.30	1.13 (0.90-1.43)	1.11 (0.88-1.40)	97	0.40	1.47 (1.20-1.80)	1.33 (1.09-1.63)
Severe	28	0.40	1.74 (1.20-2.52)	1.74 (1.20-2.52)	34	0.49	2.22 (1.58-3.10)	1.91 (1.36-2.67)
**Preeclampsia with/without SGA**								
Without SGA	62	0.27	1.16 (0.90-1.49)	1.11 (0.86-1.43)	86	0.38	1.61 (1.30-1.99)	1.41 (1.14-1.74)
With SGA	30	0.42	1.80 (1.26-2.58)	1.74 (1.22-2.50)	36	0.50	2.18 (1.57-3.02)	1.90 (1.37-2.64)

© 2022 Yang F et al. *JAMA Network Open.*

Other hypertensive disorders during pregnancy								
Pre-existing chronic hypertension	15	0.35	1.80 (1.08-2.99)	1.82 (1.10-3.04)	15	0.35	1.86 (1.12-3.10)	1.44 (0.87-2.40)
Gestational hypertension	45	0.29	1.17 (0.87-1.56)	1.17 (0.87-1.57)	67	0.43	1.71 (1.34-2.18)	1.55 (1.22-1.97)

Abbreviations: SGA, small for gestational age (i.e., birth weight below the 10th percentile of the Swedish sex-specific standard curve for normal fetal growth); HR, hazard ratio; CI, confidence interval.

[a] We adjusted for calendar year of birth, sex, maternal parity, age, education, marital status, and diabetes before childbirth.

© 2022 Yang F et al. *JAMA Network Open.*

eTable 7. Incidence rates and hazard ratios with 95% confidence intervals for ischemic heart disease and stroke according to preeclampsia, stratified by study country

Exposure	Number of events	Rate, per 10,000 person-years	Crude HR (95% CI)	Adjusted HR (95% CI) [a]	P-value for interaction
**Ischemic heart disease [b]**					
**Denmark**					
Normotensive pregnancy	4,972	0.90	1.00	1.00	
Preeclampsia	98	0.68	1.11 (0.91-1.36)	1.09 (0.89-1.33)	
**Sweden**					0.92
Normotensive pregnancy	1,457	0.18	1.00	1.00	
Preeclampsia	16	0.13	1.45 (0.88-2.37)	1.37 (0.83-2.25)	
**Finland**					0.28
Normotensive pregnancy	808	0.37	1.00	1.00	
Preeclampsia	19	0.43	1.10 (0.70-1.73)	1.14 (0.72-1.79)	
**Stroke [b]**					
**Denmark**					
Normotensive pregnancy	5,357	0.97	1.00	1.00	
Preeclampsia	144	1.00	1.26 (1.07-1.48)	1.22 (1.04-1.44)	
**Sweden**					0.86
Normotensive pregnancy	4,250	0.52	1.00	1.00	
Preeclampsia	63	0.50	1.36 (1.06-1.74)	1.24 (0.97-1.59)	
**Finland**					0.71
Normotensive pregnancy	938	0.43	1.00	1.00	
Preeclampsia	26	0.58	1.32 (0.89-1.95)	1.32 (0.89-1.94)	

Abbreviations: HR, hazard ratio; CI, confidence interval.

[a] We adjusted for calendar year of birth, sex, maternal parity, age, education, marital status, and diabetes before childbirth.

© 2022 Yang F et al. *JAMA Network Open.*

eTable 8. Incidence rates and hazard ratios with 95% confidence intervals for ischemic heart disease and stroke according to preeclampsia, stratified by the child's sex

Exposure	Number of events	Rate, per 10,000 person-years	Crude HR (95% CI)	Adjusted HR (95% CI) [a]	P-value for interaction
**Ischemic heart disease [b]**					
**Boys**					0.42
Normotensive pregnancy	4,640	0.57	1.00	1.00	
Preeclampsia	80	0.49	1.33 (1.06-1.66)	1.26 (1.01-1.57)	
**Girls**					
Normotensive pregnancy	2,597	0.34	1.00	1.00	
Preeclampsia	53	0.35	1.53 (1.17-2.01)	1.47 (1.12-1.93)	
**Stroke [b]**					
**Boys**					0.95
Normotensive pregnancy	5,493	0.67	1.00	1.00	
Preeclampsia	124	0.76	1.36 (1.13-1.62)	1.32 (1.11-1.58)	
**Girls**					
Normotensive pregnancy	5,052	0.65	1.00	1.00	
Preeclampsia	109	0.72	1.38 (1.14-1.67)	1.35 (1.11-1.63)	

Abbreviations: HR, hazard ratio; CI, confidence interval.

[a] We adjusted for calendar year of birth, maternal parity, age, education, marital status, and diabetes before childbirth.

© 2022 Yang F et al. *JAMA Network Open.*

eTable 9. Incidence rates and hazard ratios with 95% confidence intervals for ischemic heart disease and stroke according to preeclampsia and its subtypes, in children without congenital anomalies

Exposure	Ischemic heart disease				Stroke			
	Number of events	Rate, per 10,000 person-years	Crude HR (95% CI)	Adjusted HR (95% CI) [a]	Number of events	Rate, per 10,000 person-years	Crude HR (95% CI)	Adjusted HR (95% CI) [a]
**Normotensive pregnancy**	5,940	0.41	1.00	1.00	8,001	0.55	1.00	1.00
**Preeclampsia**	96	0.35	1.35 (1.10-1.65)	1.28 (1.04-1.57)	155	0.56	1.28 (1.09-1.50)	1.25 (1.06-1.46)
**Onset of preeclampsia**								
Late onset	66	0.29	1.31 (1.03-1.68)	1.25 (0.98-1.60)	106	0.47	1.14 (0.94-1.38)	1.12 (0.92-1.35)
Early onset	13	0.34	1.73 (1.01-2.98)	1.62 (0.94-2.79)	36	0.94	2.38 (1.72-3.31)	2.32 (1.67-3.22)
**Severity of preeclampsia**								
Mild/moderate	78	0.36	1.32 (1.06-1.65)	1.24 (0.99-1.56)	114	0.53	1.16 (0.97-1.40)	1.13 (0.94-1.36)
Severe	18	0.30	1.46 (0.92-2.32)	1.45 (0.91-2.31)	41	0.68	1.75 (1.29-2.38)	1.74 (1.28-2.36)
**Preeclampsia with/without SGA**								
Without SGA	58	0.29	1.51 (1.16-1.95)	1.23 (0.95-1.59)	102	0.51	1.31 (1.08-1.60)	1.20 (0.99-1.46)
With SGA	21	0.35	1.87 (1.22-2.87)	1.58 (1.03-2.43)	39	0.64	1.69 (1.24-2.32)	1.56 (1.14-2.14)
**Other hypertensive disorders during pregnancy**								
Pre-existing chronic hypertension	7	0.18	1.23 (0.58-2.58)	1.21 (0.57-2.54)	23	0.60	1.73 (1.15-2.61)	1.77 (1.18-2.68)
Gestational hypertension	54	0.38	1.59 (1.21-2.08)	1.58 (1.21-2.07)	80	0.57	1.32 (1.06-1.65)	1.34 (1.07-1.67)

Abbreviations: SGA, small for gestational age (i.e., birth weight below the 10th percentile of the Swedish sex-specific standard curve for normal fetal growth); HR, hazard ratio; CI, confidence interval.
[a] We adjusted for calendar year of birth, sex, maternal parity, age, education, marital status, and diabetes before childbirth.

© 2022 Yang F et al. *JAMA Network Open.*

eTable 10. Incidence rates and hazard ratios with 95% confidence intervals for ischemic heart disease and stroke according to preeclampsia and its subtypes, in children with information on maternal country of origin

Exposure	Ischemic heart disease				Stroke			
	Rate, per 10,000 person-years	Adjusted HR in the whole population (95% CI) [a]	Adjusted HR in the restricted population (95% CI) [b]	Adjusted HR in the restricted population (95% CI) [c]	Rate, per 10,000 person-years	Adjusted HR in the whole population (95% CI) [a]	Adjusted HR in the restricted population (95% CI) [b]	Adjusted HR in the restricted population (95% CI) [c]
**Normotensive pregnancy**	0.47	1.00	1.00	1.00	0.70	1.00	1.00	1.00
**Preeclampsia**	0.42	1.33 (1.12-1.58)	1.43 (1.19-1.73)	1.44 (1.20-1.74)	0.77	1.34 (1.17-1.52)	1.34 (1.17-1.54)	1.34 (1.16-1.54)
**Onset of preeclampsia**								
Late onset	0.36	1.31 (1.06-1.61)	1.48 (1.18-1.85)	1.48 (1.18-1.85)	0.66	1.18 (1.01-1.39)	1.22 (1.03-1.44)	1.22 (1.03-1.44)
Early onset	0.36	1.62 (1.03-2.55)	1.66 (0.98-2.81)	1.68 (0.99-2.84)	1.27	2.55 (1.97-3.28)	2.45 (1.85-3.24)	2.45 (1.85-3.24)
**Severity of preeclampsia**								
Mild/moderate	0.47	1.33 (1.10-1.61)	1.46 (1.17-1.83)	1.47 (1.20-1.80)	0.77	1.22 (1.05-1.42)	1.28 (1.09-1.49)	1.27 (1.09-1.49)
Severe	0.27	1.33 (0.89-2.01)	1.29 (0.79-2.11)	1.29 (0.79-2.11)	0.84	1.81 (1.41-2.32)	1.60 (1.21-2.13)	1.60 (1.21-2.13)
**Preeclampsia with/without SGA**								
Without SGA	0.35	1.32 (1.06-1.64)	1.41 (1.10-1.80)	1.42 (1.11-1.81)	0.69	1.25 (1.07-1.48)	1.30 (1.09-1.54)	1.29 (1.09-1.54)
With SGA	0.42	1.50 (1.03-2.18)	1.81 (1.22-2.69)	1.82 (1.23-2.70)	0.93	1.84 (1.44-2.34)	1.78 (1.36-2.31)	1.78 (1.36-2.31)
**Other hypertensive disorders during pregnancy**								
Pre-existing chronic hypertension	0.37	1.72 (1.02-2.92)	2.24 (1.20-4.18)	2.27 (1.22-4.22)	0.78	1.60 (1.12-2.30)	1.64 (1.07-2.52)	1.64 (1.07-2.53)
Gestational hypertension	0.44	1.44 (1.12-1.84)	1.44 (1.08-1.94)	1.45 (1.08-1.95)	0.86	1.40 (1.16-1.68)	1.45 (1.18-1.79)	1.45 (1.17-1.79)

Abbreviations: SGA, small for gestational age (i.e., birth weight below the 10th percentile of the Swedish sex-specific standard curve for normal fetal growth); HR, hazard ratio; CI, confidence interval.

© 2022 Yang F et al. *JAMA Network Open.*

[a] In the whole population, we adjusted for calendar year of birth, sex, maternal parity, age, education, marital status, and diabetes before childbirth.
[b] In study participants with information on maternal country of origin, we adjusted for calendar year of birth, sex, maternal parity, age, education, marital status, and diabetes before childbirth.
[c] In study participants with information on maternal country of origin, we further adjusted for maternal country of origin.

© 2022 Yang F et al. *JAMA Network Open.*

eTable 11. Incidence rates and hazard ratios with 95% confidence intervals for ischemic heart disease and stroke according to preeclampsia and its subtypes, in children with information on maternal smoking during early pregnancy

Exposure	Ischemic heart disease				Stroke			
	Rate, per 10,000 person-years	Adjusted HR in the whole population (95% CI) [a]	Adjusted HR in the restricted population (95% CI) [b]	Adjusted HR in the restricted population (95% CI) [c]	Rate, per 10,000 person-years	Adjusted HR in the whole population (95% CI) [a]	Adjusted HR in the restricted population (95% CI) [b]	Adjusted HR in the restricted population (95% CI) [c]
**Normotensive pregnancy**	0.17	1.00	1.00	1.00	0.41	1.00	1.00	1.00
**Preeclampsia**	0.22	1.33 (1.12-1.58)	1.20 (0.90-1.62)	1.23 (0.92-1.65)	0.53	1.34 (1.17-1.52)	1.28 (1.06-1.54)	1.29 (1.07-1.56)
**Onset of preeclampsia**								
Late onset	0.21	1.31 (1.06-1.61)	1.18 (0.86-1.63)	1.21 (0.88-1.68)	0.45	1.18 (1.01-1.39)	1.07 (0.86-1.34)	1.09 (0.87-1.36)
Early onset	0.23	1.62 (1.03-2.55)	1.37 (0.68-2.75)	1.38 (0.69-2.76)	0.99	2.55 (1.97-3.28)	2.36 (1.68-3.31)	2.37 (1.69-3.32)
**Severity of preeclampsia**								
Mild/moderate	0.23	1.33 (1.10-1.61)	1.25 (0.90-1.75)	1.28 (0.92-1.78)	0.47	1.22 (1.05-1.42)	1.11 (0.88-1.40)	1.12 (0.89-1.41)
Severe	0.18	1.33 (0.89-2.01)	1.05 (0.56-1.96)	1.09 (0.58-2.02)	0.73	1.81 (1.41-2.32)	1.77 (1.30-2.42)	1.80 (1.32-2.46)
**Preeclampsia with/without SGA**								
Without SGA	0.24	1.32 (1.06-1.64)	1.33 (0.96-1.84)	1.37 (0.99-1.89)	0.45	1.25 (1.07-1.48)	1.06 (0.84-1.34)	1.08 (0.85-1.36)
With SGA	0.15	1.50 (1.03-2.18)	0.88 (0.44-1.75)	0.88 (0.44-1.77)	0.83	1.84 (1.44-2.34)	1.98 (1.46-2.67)	1.99 (1.47-2.68)
**Other hypertensive disorders during pregnancy**								
Pre-existing chronic hypertension	0.26	1.72 (1.02-2.92)	1.66 (0.89-3.10)	1.68 (0.90-3.14)	0.52	1.60 (1.12-2.30)	1.28 (0.82-1.99)	1.29 (0.83-2.01)
Gestational hypertension	0.24	1.44 (1.12-1.84)	1.34 (0.92-1.94)	1.36 (0.93-1.97)	0.63	1.40 (1.16-1.68)	1.49 (1.18-1.88)	1.50 (1.19-1.89)

Abbreviations: SGA, small for gestational age (i.e., birth weight below the 10th percentile of the Swedish sex-specific standard curve for normal fetal growth); HR, hazard ratio; CI, confidence interval.

© 2022 Yang F et al. *JAMA Network Open.*

[a] In the whole population, we adjusted for calendar year of birth, sex, maternal parity, age, education, marital status, and diabetes before childbirth.
[b] In study participants with information on maternal smoking during pregnancy, we adjusted for calendar year of birth, sex, maternal parity, age, education, marital status, and diabetes before childbirth.
[c] In study participants with information on maternal smoking during pregnancy, we further adjusted for maternal smoking during early pregnancy.

© 2022 Yang F et al. *JAMA Network Open.*

eTable 12. Incidence rates and hazard ratios with 95% confidence intervals for ischemic heart disease and stroke according to preeclampsia and its subtypes, in children with information on maternal body mass index during early pregnancy

Exposure	Ischemic heart disease				Stroke			
	Rate, per 10,000 person-years	Adjusted HR in the whole population (95% CI) [a]	Adjusted HR in the restricted population (95% CI) [b]	Adjusted HR in the restricted population (95% CI) [c]	Rate, per 10,000 person-years	Adjusted HR in the whole population (95% CI) [a]	Adjusted HR in the restricted population (95% CI) [b]	Adjusted HR in the restricted population (95% CI) [c]
**Normotensive pregnancy**	0.09	1.00	1.00	1.00	0.41	1.00	1.00	1.00
**Preeclampsia**	0.10	1.33 (1.12-1.58)	1.24 (0.66-2.33)	1.21 (0.64-2.27)	0.57	1.34 (1.17-1.52)	1.38(1.06-1.80)	1.35 (1.04-1.76)
**Onset of preeclampsia**								
Late onset	0.10	1.31 (1.06-1.61)	1.28 (0.66-2.48)	1.24 (0.64-2.42)	0.50	1.18 (1.01-1.39)	1.22 (0.91-1.65)	1.20 (0.89-1.62)
Early onset	0.08	1.62 (1.03-2.55)	0.98 (0.14-7.01)	0.96 (0.13-6.84)	1.10	2.55 (1.97-3.28)	2.34 (1.38-3.97)	2.29 (1.35-3.88)
**Severity of preeclampsia**								
Mild/moderate	0.11	1.33 (1.10-1.61)	1.33 (0.66-2.69)	1.29 (0.64-2.61)	0.57	1.22 (1.05-1.42)	1.37 (1.01-1.86)	1.34 (0.99-1.83)
Severe	0.07	1.33 (0.89-2.01)	0.98 (0.24-3.93)	0.96 (0.24-3.84)	0.58	1.81 (1.41-2.32)	1.41 (0.86-2.30)	1.38 (0.84-2.26)
**Preeclampsia with/without SGA**								
Without SGA	0.08	1.32 (1.06-1.64)	0.97 (0.43-2.17)	0.94 (0.42-2.11)	0.49	1.25 (1.07-1.48)	1.17 (0.85-1.63)	1.14 (0.82-1.59)
With SGA	0.17	1.50 (1.03-2.18)	2.12 (0.79-5.69)	2.09 (0.78-5.60)	0.87	1.84 (1.44-2.34)	2.11 (1.37-3.24)	2.07 (1.34-3.19)
**Other hypertensive disorders during pregnancy**								
Pre-existing chronic hypertension	0.30	1.72 (1.02-2.92)	3.76 (1.39-10.14)	3.64 (1.35-9.84)	0.52	1.60 (1.12-2.30)	1.14 (0.54-2.40)	1.10 (0.52-2.33)
Gestational hypertension	0.14	1.44 (1.12-1.84)	1.62 (0.72-3.62)	1.57 (0.70-3.53)	0.72	1.40 (1.16-1.68)	1.63 (1.14-2.34)	1.60 (1.11-2.30)

Abbreviations: SGA, small for gestational age (i.e., birth weight below the 10th percentile of the Swedish sex-specific standard curve for normal fetal growth); HR, hazard ratio; CI, confidence interval..

© 2022 Yang F et al. *JAMA Network Open.*

[a] In the whole population, we adjusted for calendar year of birth, sex, maternal parity, age, education, marital status, and diabetes before childbirth.
[b] In study participants with information on maternal body mass index during pregnancy, we adjusted for calendar year of birth, sex, maternal parity, age, education, marital status, and diabetes before childbirth.
[c] In study participants with information on maternal body mass index during pregnancy, we further adjusted for maternal body mass index during early pregnancy.

© 2022 Yang F et al. *JAMA Network Open.*

eTable 13. Incidence rates and hazard ratios with 95% confidence intervals for ischemic heart disease and stroke according to preeclampsia and its subtypes, in children with information on maternal family history of cardiovascular diseases

Exposure	Ischemic heart disease				Stroke			
	Rate, per 10,000 person-years	Adjusted HR in the whole population (95% CI) [a]	Adjusted HR in the restricted population (95% CI) [b]	Adjusted HR in the restricted population (95% CI) [c]	Rate, per 10,000 person-years	Adjusted HR in the whole population (95% CI) [a]	Adjusted HR in the restricted population (95% CI) [b]	Adjusted HR in the restricted population (95% CI) [c]
**Normotensive pregnancy**	0.47	1.00	1.00	1.00	0.70	1.00	1.00	1.00
**Preeclampsia**	0.42	1.33 (1.12-1.58)	1.43 (1.19-1.72)	1.43 (1.19-1.73)	0.77	1.34 (1.17-1.52)	1.34 (1.16-1.54)	1.34 (1.16-1.54)
**Onset of preeclampsia**								
Late onset	0.36	1.31 (1.06-1.61)	1.46 (1.17-1.83)	1.46 (1.17-1.83)	0.66	1.18 (1.01-1.39)	1.22 (1.03-1.44)	1.22 (1.03-1.62)
Early onset	0.36	1.62 (1.03-2.55)	1.66 (0.98-2.80)	1.66 (0.98-2.81)	1.27	2.55 (1.97-3.28)	2.45 (1.85-3.24)	2.44 (1.85-3.23)
**Severity of preeclampsia**								
Mild/moderate	0.47	1.33 (1.10-1.61)	1.46 (1.19-1.78)	1.46 (1.19-1.78)	0.76	1.22 (1.05-1.42)	1.27 (1.09-1.49)	1.27 (1.09-1.49)
Severe	0.27	1.33 (0.89-2.01)	1.29 (0.79-2.10)	1.29 (0.79-2.11)	0.80	1.81 (1.41-2.32)	1.60 (1.21-2.13)	1.60 (1.21-2.13)
**Preeclampsia with/without SGA**								
Without SGA	0.35	1.32 (1.06-1.64)	1.40 (1.10-1.79)	1.40 (1.10-1.79)	0.69	1.25 (1.07-1.48)	1.29 (1.09-1.54)	1.29 (1.09-1.54)
With SGA	0.42	1.50 (1.03-2.18)	1.81 (1.22-2.68)	1.81 (1.22-2.68)	0.93	1.84 (1.44-2.34)	1.77 (1.36-2.31)	1.77 (1.36-2.31)
**Other hypertensive disorders during pregnancy**								
Pre-existing chronic hypertension	0.37	1.72 (1.02-2.92)	2.23 (1.20-4.16)	2.27 (1.22-4.24)	0.82	1.60 (1.12-2.30)	1.72 (1.13-2.62)	1.73 (1.14-2.63)
Gestational hypertension	0.44	1.44 (1.12-1.84)	1.44 (1.07-1.93)	1.45 (1.08-1.94)	0.86	1.40 (1.16-1.68)	1.46 (1.18-1.80)	1.46 (1.18-1.80)

Abbreviations: SGA, small for gestational age (i.e., birth weight below the 10th percentile of the Swedish sex-specific standard curve for normal fetal growth); HR, hazard ratio; CI, confidence interval.

© 2022 Yang F et al. *JAMA Network Open.*

[a] In the whole population, we adjusted for calendar year of birth, sex, maternal parity, age, education, marital status, and diabetes before childbirth.
[b] In study participants with information on maternal family history of cardiovascular diseases, we adjusted for calendar year of birth, sex, maternal parity, age, education, marital status, and diabetes before childbirth.
[c] In study participants with information on maternal family history of cardiovascular diseases, we further adjusted for family history of cardiovascular diseases.

© 2022 Yang F et al. *JAMA Network Open.*

eFigure. Cumulative incidence of A, ischemic heart disease, and B, stroke according to preeclampsia

A

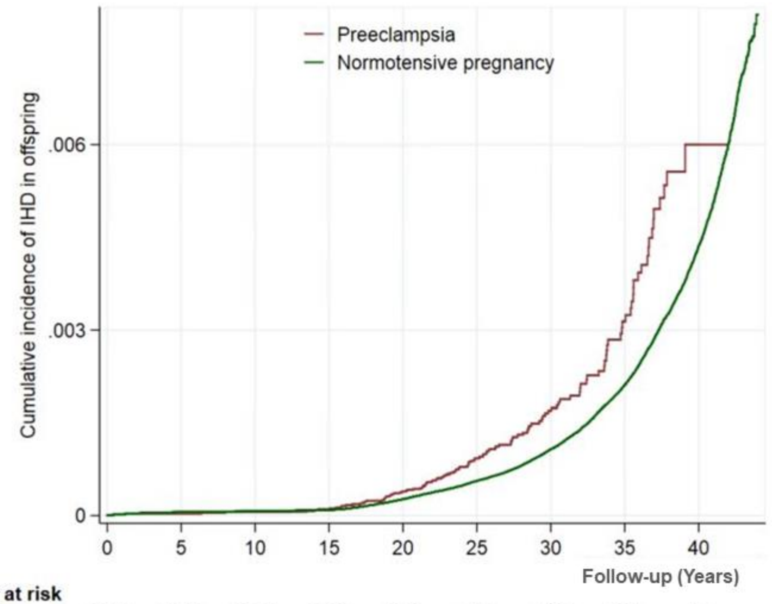

Number at risk									
Preeclampsia	188670	155667	126882	101064	75309	45041	22252	9700	423
Normotensive pregnancy	8147974	6976437	5907014	4922833	3922788	2729257	1774292	1115966	437043

B

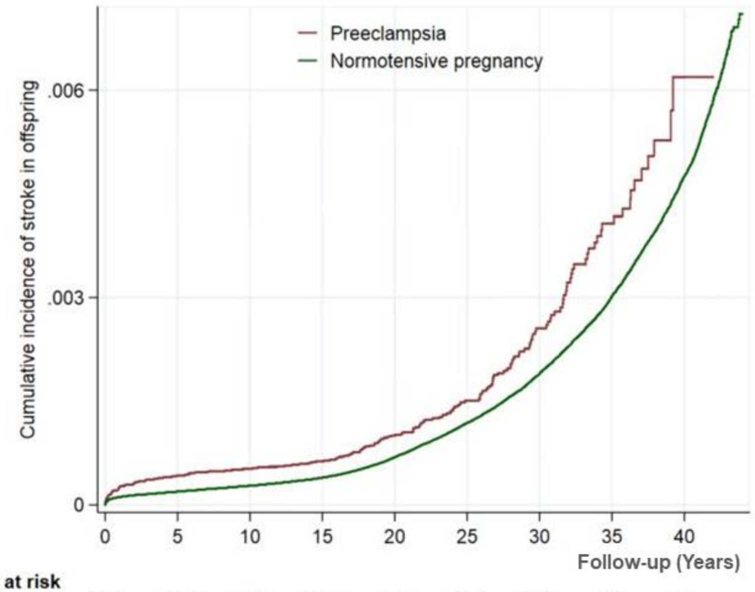

Number at risk									
Preeclampsia	188498	155625	126845	101032	75275	45018	22241	9701	424
Normotensive pregnancy	8142627	6975693	5906135	4921736	3921455	2727784	1773005	1115125	437120

© 2022 Yang F et al. *JAMA Network Open.*
